# Supplementary material for: Analysis of Phenolic Components and Related Biological Activities of 35 Apple (Malus pumila Mill.) Cultivars
Source: Molecules. 2020 Sep 10;25(18):4153. doi: 10.3390/molecules25184153 (PMC7571092; doi:10.3390/molecules25184153)
Supplement: Supplementary file 1 [file molecules-25-04153-s001.zip › supplementary/Figure S2.pdf]

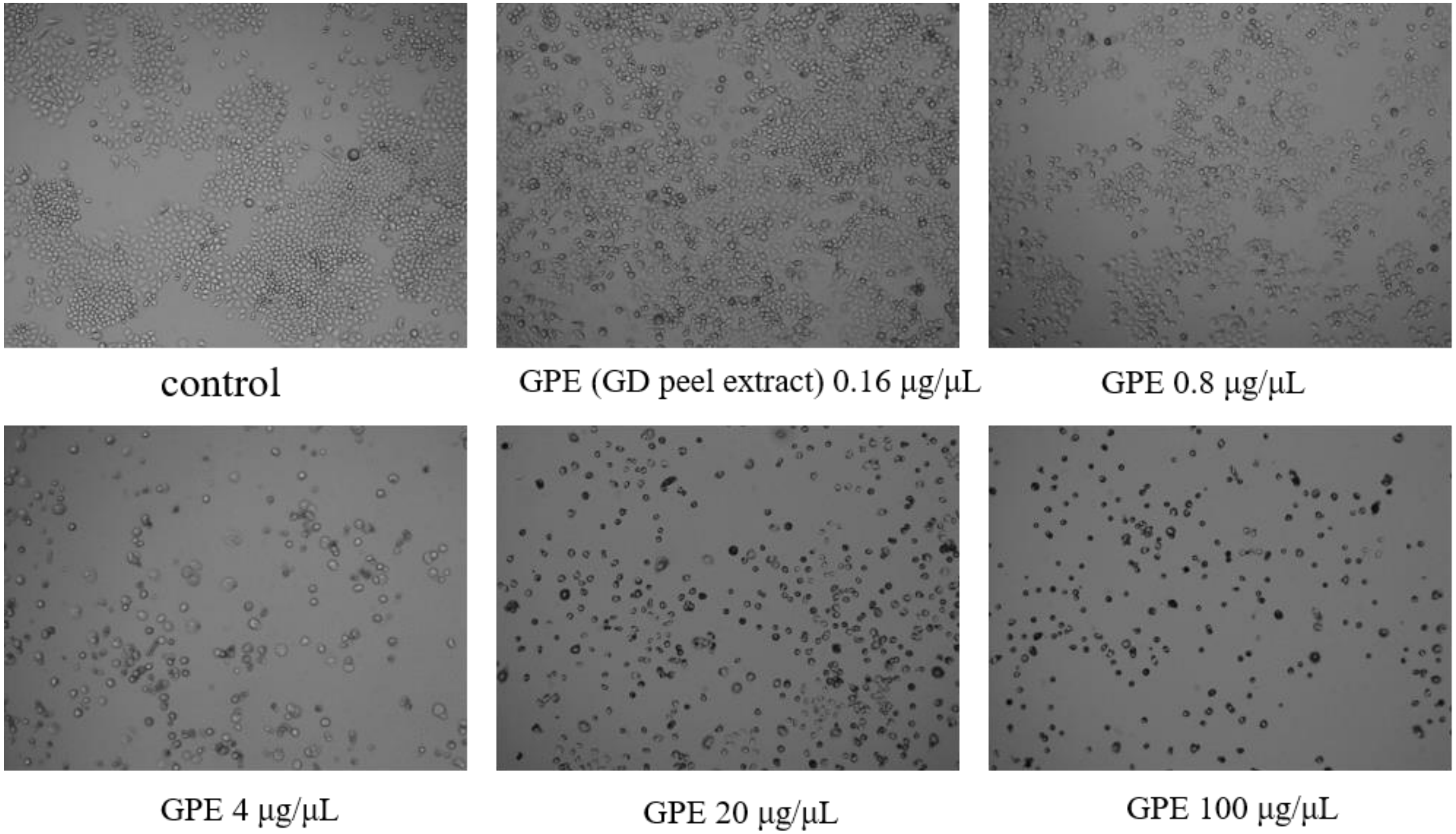

Figure S2. Photomicrographs of HepG2 cells treated with different concentrations of apple extract (take GD peel extract as an example).
