# Supplementary material for: Analysis of Phenolic Components and Related Biological Activities of 35 Apple (Malus pumila Mill.) Cultivars
Source: Molecules. 2020 Sep 10;25(18):4153. doi: 10.3390/molecules25184153 (PMC7571092; doi:10.3390/molecules25184153)
Supplement: Supplementary file 1 [file molecules-25-04153-s001.zip › supplementary/Table S1 Apple cultivars used in present study.pdf]

Table S1. Apple cultivars used in present study

Classification	Cultivar
Early maturing cultivars	Vista Bella
	Mato
	Early McIntosh
	Tianyisayewa
	Chunxiang
	Faxian
	Matsumoto Nishiki
	Babusijinuo
	Xiboliyabaidian
	Basimei
	Honglu
	Nuoda
Medium maturing cultivars	Bolan No.8
	Southern Snap
	Qianqiu
	Gala
	Hermhut
	Ximengfei
	Sakata Tsugaru
	Yingqiu
	Mantanghong
	Qiuying
Late maturing cultivars	Fuju
	Gold Delicious
	Jonagold
	Jingxiang
	Xinshijie
	Danguang
	Dounan
	Huahong
	Yuehong
	New Jonagold
	Rizhiwan
	Hanfu
	Huaguan
